# Zika Virus, French Polynesia, South Pacific, 2013

**DOI:** 10.3201/eid2011.141253

**Published:** 2014-11

**Authors:** W. Thane Hancock, Maria Marfel, Martin Bel

**Affiliations:** Yap State Department of Health Services, Colonia, Yap, Federated States of Micronesia

**Keywords:** Zika virus, ZIKV, viruses, outbreak, dengue, dengue virus, Yap, Micronesia

**To the Editor:** We wish to clarify an inaccuracy in a letter in Emerging Infectious Diseases by Cao-Lormeau et al. (*1*). The authors state “In 2007, the first ZIKV outbreak reported outside Africa and Asia was retrospectively documented from biological samples from patients on Yap Island, Federated States of Micronesia, North Pacific, who had received an incorrect diagnosis of dengue virus (DENV).” Although the first outbreak of Zika virus (ZIKV) infection reported outside Africa or Asia was in Yap, it was not retrospectively identified from serum samples incorrectly diagnosed as positive for dengue virus. The outbreak was first identified by the Yap State Department of Health Services, and an investigation to determine the etiologic agent was initiated.

Although dengue was initially part of the differential diagnosis, and a few patients had evidence of IgM against dengue virus by a rapid diagnostic test, clinicians in Yap believed that the clinical syndrome was not consistent with dengue. Thus, assistance was requested from the US Centers for Disease Control and Prevention and the World Health Organization to strengthen the epidemiologic investigation and provide confirmatory laboratory testing.

Serum samples collected during the active investigation were sent to the Arboviral Diseases Diagnostic Laboratory at the Centers for Disease Control and Prevention where testing determined that the cause of the infections was ZIKV (*2*). This discovery of ZIKV as the etiologic agent was not achieved through retrospective testing of serum from patients incorrectly diagnosed as having dengue, but rather the result of an active, coordinated investigation by the Yap State Department of Health Services with instrumental assistance from international partners.
